# Breast cancer subtype dictates DNA methylation and ALDH1A3-mediated expression of tumor suppressor RARRES1

**DOI:** 10.18632/oncotarget.9858

**Published:** 2016-06-06

**Authors:** Krysta M. Coyle, J. Patrick Murphy, Dejan Vidovic, Ahmad Vaghar-Kashani, Cheryl A. Dean, Mohammad Sultan, Derek Clements, Melissa Wallace, Margaret L. Thomas, Amos Hundert, Carman A. Giacomantonio, Lucy Helyer, Shashi A. Gujar, Patrick W.K. Lee, Ian C.G. Weaver, Paola Marcato

**Affiliations:** ^1^ Department of Pathology, Dalhousie University, Halifax, NS, Canada; ^2^ Department of Microbiology & Immunology, Dalhousie University, Halifax, NS, Canada; ^3^ Department of Biology Education Center, Uppsala University, Uppsala, Sweden; ^4^ Department of Psychology and Neuroscience, and Psychiatry, Dalhousie University, Halifax, NS, Canada; ^5^ Department of Surgery, Dalhousie University, Halifax, NS, Canada; ^6^ Department of Quality and System Performance, IWK Health Centre, Halifax, NS, Canada

**Keywords:** breast cancer, RARRES1, retinoic acid, ALDH1A3, DNA methylation

## Abstract

Breast cancer subtyping, based on the expression of hormone receptors and other genes, can determine patient prognosis and potential options for targeted therapy. Among breast cancer subtypes, tumors of basal-like and claudin-low subtypes are typically associated with worse patient outcomes, are primarily classified as triple-negative breast cancers (TNBC), and cannot be treated with existing hormone-receptor-targeted therapies. Understanding the molecular basis of these subtypes will lead to the development of more effective treatment options for TNBC. In this study, we focus on retinoic acid receptor responder 1 (RARRES1) as a paradigm to determine if breast cancer subtype dictates protein function and gene expression regulation. Patient tumor dataset analysis and gene expression studies of a 26 cell-line panel, representing the five breast cancer subtypes, demonstrate that RARRES1 expression is greatest in basal-like TNBCs. Cell proliferation and tumor growth assays reveal that RARRES1 is a tumor suppressor in TNBC. Furthermore, gene expression studies, Illumina HumanMethylation450 arrays, and chromatin immunoprecipitation demonstrate that expression of RARRES1 is retained in basal-like breast cancers due to hypomethylation of the promoter. Additionally, expression of the cancer stem cell marker, aldehyde dehydrogenase 1A3, which provides the required ligand (retinoic acid) for RARRES1 transcription, is also specific to the basal-like subtype. We functionally demonstrate that the combination of promoter methylation and retinoic acid signaling dictates expression of tumor suppressor RARRES1 in a subtype-specific manner. These findings provide a precedent for a therapeutically-inducible tumor suppressor and suggest novel avenues of therapeutic intervention for patients with basal-like breast cancer.

## INTRODUCTION

Although mortality from breast cancer has significantly declined over the past 20 years, breast cancer remains a leading cause of death for women around the world [1]. Novel therapeutic strategies are required to continue making strides against this prevalent disease. Breast cancer has five major molecular subtypes; luminal A/B, HER2 positive, basal-like, and claudin-low. Luminal A/B breast cancers typically express the estrogen receptor (ER) and progesterone receptor (PR), while Her2-like are typically characterized by overexpression of the human epidermal growth factor receptor (ERBB2, Her2/neu) [2]. Expression of these receptors allows for treatment with hormone receptor antagonist therapies (e.g. tamoxifen), which have vastly improved the survival of breast cancer patients with hormone-receptor-positive tumors [3]. This is in contrast to basal-like and claudin-low tumors, which are predominately hormone receptor negative (triple-negative breast cancer; TNBC) and are not treatable by hormone receptor antagonists. Patients with claudin-low or basal-like TNBCs have poorer outcomes with a greater likelihood of metastasis development and more limited treatment options.

Adding to the complexity of patient outcomes, basal-like and claudin-low subtypes differ with respect to prognosis and can be stratified by gene copy-number alterations, genomic instability, gene expression profiles, and distinct drug sensitivities [4]. It is almost certain that differences in the genes expressed in these tumors are responsible for their responses to select agents. Understanding the molecular basis of these breast cancer subtypes will lead to the development of more effective treatment options for TNBC.

Genes can be identified as correlative (e.g. biomarkers) or causative (oncogenes or tumor suppressors) when examining the response of breast cancer subtypes to different therapies. Several approaches exist to identify causative genes. First, mutations and epigenetic modifications can affect the expression of genes such that their role as a tumor suppressor or oncogene is amplified or diminished in different subtypes. DNA methylation is one of the most studied mechanisms affecting gene expression: changes in methylation in many human diseases have been reported, with over 20 000 papers describing these alterations in cancer. Within these studies, several reports have identified promoter-associated hypermethylation in the context of genomic hypomethylation in breast cancer tissue when compared to normal or benign lesions [5,6]. Additional studies have observed subtype-specific methylation patterns [7–11]; and several tumor-promoting and tumor-suppressing genes have already been identified as differentially methylated in breast cancer subtypes, affecting their expression [12,13]. Second, genes and gene products may have different roles and functions depending on their cellular context. For example, the androgen receptor has been suggested as a tumor suppressor in ER-positive tumors, while playing an oncogenic role in ER-negative tumors [14]. Our own work identified the cancer stem cell marker and retinoic-acid (RA) producing enzyme, aldehyde dehydrogenase 1A3 (ALDH1A3), as promoting or suppressing tumor growth in a context-dependent manner in TNBC [15]. The RA receptor responder protein 1 (RARRES1) has also been identified as either suppressing or promoting tumor growth, depending on the study [16,17].

In this study, we focus on RARRES1 as a paradigm to determine if protein function and gene expression regulation in breast cancer is dictated by subtype. Gene expression studies with a panel of 26 cell lines and analyses of patient data sets reveal that RARRES1 expression is associated with TNBCs, specific to the basal-like subtype. Cell proliferation, tumor growth assays, proteome and cellular localization studies demonstrate it acts as a tumor suppressor in TNBC. HumanMethylation450 arrays and chromatin immunoprecipitation (ChIP) analyses demonstrate that RARRES1 expression is subtype-dependent and regulated dually by DNA methylation and the expression of ALDH1A3, which produces its transcription-inducing factor, RA. These findings provide a precedent for a therapeutically-inducible tumor suppressor and suggest potential avenues of therapeutic intervention for TNBC patients who lack targeted therapies.

## RESULTS

### Basal-like breast cancer tumors express higher levels of RARRES1

**Figure 1:**
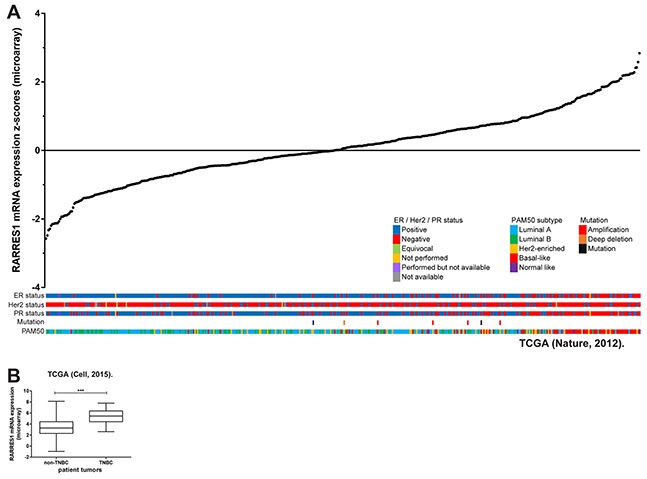
RARRES1 is highly expressed in triple-negative breast cancer **A.** Expression of RARRES1 mRNA was obtained from cBioportal [18] and plotted in ascending order with corresponding ER / Her2 / PR status, mutation status, and PAM50 subtype. Samples without mRNA expression data are listed separately. **B.** Expression of RARRES1 mRNA was obtained from cBioportal [31] and plotted by TNBC / non-TNBC.

To investigate if RARRES1 represents a gene that is differentially expressed in the molecular subtypes of breast cancer, we obtained data from the 2012 TCGA breast cancer data set [18] using the cBioportal interface [19,20]. The arrangement of RARRES1 expression in individual tumors allowed us to identify that ER-, PR- and Her2-negative status was associated with higher RARRES1 expression. Additionally, the data set was examined for PAM50 subtype which allowed separation into the luminal A/B, Her2-enriched, and basal-like subtypes based on expression of 50 genes (PAM50 classification does not include the claudin-low subtype). High expression of RARRES1 was associated with the basal-like subtype (Figure 1A).

Since TNBCs are primarily basal-like (50% of TNBCs) [21], we expected that RARRES1 would also be higher in TNBC tumors. We separated the 2015 TCGA data set using the cBioportal interface for TNBC and non-TNBC tumors. RARRES1 expression was significantly higher in the TNBC tumors (Figure 1B). From this data analysis, it is unclear how many of the high RARRES1-expressing TNBC are claudin-low (approximately 30% of TNBC are claudin-low) [21], and if the association of RARRES1 with TNBCs is specific to either the basal-like or claudin-low subtype.

**Figure 2:**
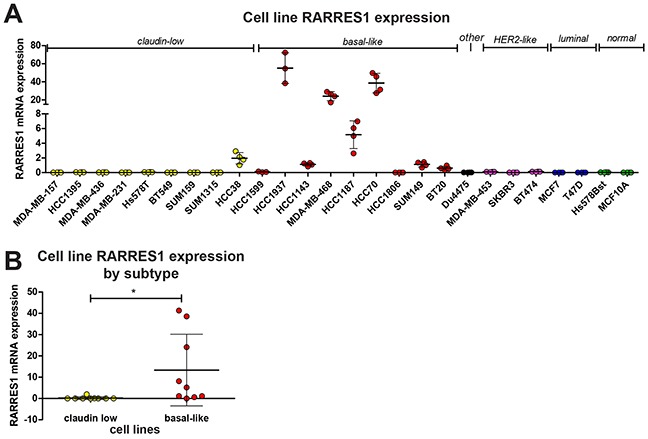
RARRES1 is highly expressed in basal-like cell lines **A.** RARRES1 expression in 24 cancerous and 2 normal breast cell lines was determined by qPCR; **B.** the mean value from each claudin low and basal-like cell line were plotted and compared by a student's t-test. For all statistical comparisons, * p<0.05, ** p<0.01, *** p<0.001.

To answer this question and validate these findings, we selected 24 cell lines which have been previously characterized as claudin-low, basal-like, Her2-like, luminal, or other (Supplementary Table S1), as well as two immortalized normal breast cell lines (Hs78Bst and MCF-10A). This series includes 20 TNBC cell lines, with representation of both claudin-low and basal-like TNBCs, and would allow us to confirm that RARRES1 expression is associated with TNBC and if it is specific to the claudin-low or basal-like subtypes. Analysis of existing cell-line databases revealed no known mutations in RARRES1 [22,23], which is consistent with the low frequency of mutations in patient tumors observed in Figure 1. We quantified RARRES1 expression in these cell lines by quantitative PCR (qPCR, Figure 2A). RARRES1 was detected in all but four cell lines (SUM159, SUM1315, HCC1806, and MCF10A). While the number of Her2-like, luminal, and normal breast cell lines prohibited robust statistical analysis, the cell line data mirrored the patient data and we determined that basal-like cell lines had significantly higher mRNA expression of RARRES1 than the claudin-low cell lines (Figure 2B). We also identified significant variability of RARRES1 expression within the basal-like cell lines, which may reflect the heterogeneity known to exist within this breast cancer subtype [24]. Taken together, our data suggests that high expression of RARRES1 in TNBCs is due to the predominantly high expression of RARRES1 in the basal-like subtype, and prompted our focus in our functional assays on TNBCs.

### RARRES1 exhibits tumor suppressive effects in TNBC

**Figure 3:**
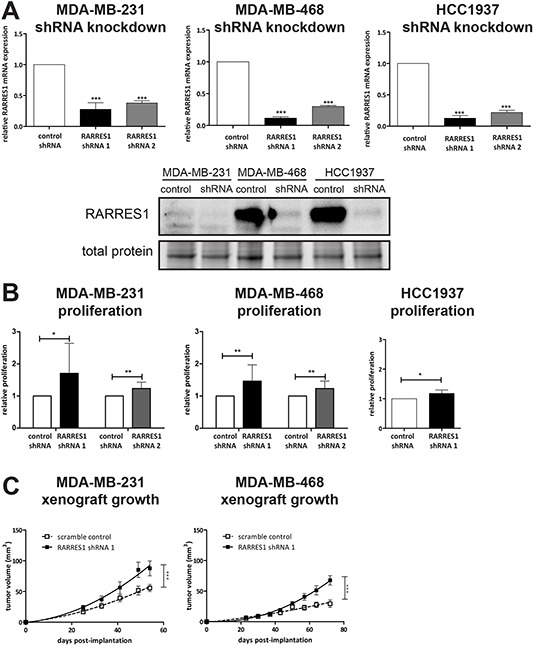
Knockdown of RARRES1 increases in vitro and in vivo cell growth **A.** shRNA knockdowns of MDA-MB-231, MDA-MB-468and HCC1937 were verified by qPCR and western blot, and compared to scramble shRNA by one-way ANOVA. **B.** The effect of RARRES1 knockdown on *in vitro* cell proliferation as compared to the scramble shRNA (by paired student's t-test). **C.** Effect of RARRES1 knockdown on tumor volume was quantified in MDA-MB-231 and MDA-MB-468 cells implanted into NOD/SCID female mice. Tumor growth was modeled using a non-linear (exponential) regression and compared by extra-sum-of-squares F test. For all statistical comparisons, * p<0.05, ** p<0.01, *** p<0.001.

RARRES1 has been reported to have tumor suppressor function in a number of cancer types [25]. These are in contrast to a functional study in the rare inflammatory subtype of breast cancer (representing less than 5% of all breast cancers), where RARRES1 was oncogenic [16]. Furthermore, given these prior reports of both tumor suppressing and oncogenic effects of RARRES1, we considered if RARRES1 expression in a breast cancer subtype influences its function. We generated lentiviral-based shRNA knockdowns of RARRES1 in claudin-low MDA-MB-231 cells, and basal-like MDA-MB-468 and HCC1937 cells. These had reduced mRNA and protein expression of RARRES1 (Figure 3A). Next, using an *in vitro* proliferation analysis, we determined that knockdown of RARRES1 with shRNA 1 increased *in vitro* proliferation in claudin-low MDA-MB-231 cells, and basal-like MDA-MB-468 and HCC1937 cells (Figure 3B). These results were confirmed using shRNA 2 in MDA-MB-231 and MDA-MB-468 cells. Additionally, the cell proliferation experiments agreed with tumor growth studies. Tumor volume (Figure 3C) and weight (Supplementary Figure S1A) of mammary fat pad-implanted MDA-MB-231 and MDA-MB-468 cells were significantly increased upon knockdown of RARRES1. The increased tumor burden did not result in increased pulmonary metastasis (MDA-MB-231, Supplementary Figure S1B; MDA-MB-468, non-metastatic and metastasis not measured). Together, these results suggest that RARRES1 has a tumor suppressing role in TNBC regardless of molecular subtype.

### Functional analysis of RARRES1

Our finding that RARRES1 has tumor suppressive effects in TNBC regardless of subtype, differs from previous findings which suggested that RARRES1 is oncogenic in inflammatory breast cancer [16]. To attempt to rectify this discrepancy, we first investigated expression of the receptor-tyrosine kinase, AXL, which has been implicated in the oncogenic role of RARRES1. We expected that AXL expression would not be affected in MDA-MB-231 and MDA-MB-468 cells as this mechanism was associated with oncogenic RARRES1. We found no difference in AXL expression following RARRES1 knockdown (Supplementary Figure S2A). This is consistent with previous findings that AXL stabilization is an oncogenic mechanism for RARRES1 [16], and with our own findings that RARRES1 is tumor suppressive in TNBC.

Alternatively, in cells of mesenchymal origin, RARRES1 is functionally involved in the tyrosination of α-tubulin [26]. We found a modest decrease in the level of detyrosinated α-tubulin when RARRES1 was depleted (Supplementary Figure S2B). To determine if this affected tubulin stability, we investigated if knockdown of RARRES1 affected the sensitivity of MDA-MB-468 to paclitaxel, which stabilizes microtubules and prevents disassembly. We found no differences in the response of the scramble shRNA-bearing and the RARRES1 shRNA-bearing cells (Supplementary Figure S2C). Therefore, at least in cells of basal-like origin, RARRES1 function appears independent of tubulin stability.

**Figure 4:**
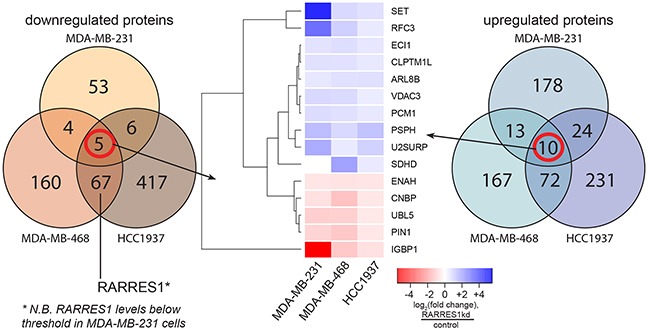
RARRES1 regulates hundreds of proteins corresponding to diverse cellular processes The effect of RARRES1 knockdown compared to scramble control on the proteome was quantified in MDA-MB-231, MDA-MB-468, and HCC1937 cells by tandem mass tag mass spectrometry of cell lysates, allowing for the detected relative protein changes. We determined a threshold for protein expression and excluded all proteins where all samples fell below the threshold. Proteins with a log_2_(fold change) > 0.379 were classified as upregulated and < −0.515 were classified as downregulated (Supplementary File 1). Upregulated and downregulated proteins visualized using a Venn diagram; 15 consistently regulated proteins were clustered using heatmap.2 (gplots, R).

The lack of changes to AXL and tubulin stability suggested the existence of other mechanisms by which RARRES1 acts as a tumor suppressor in TNBC. We performed proteomic analyses with tandem mass tag (TMT) mass spectrometry using the three TNBC cell lines where RARRES1 suppresses cell proliferation and tumor growth (MDA-MB-231, MDA-MB-468, and HCC1937, as in Figure 3) to identify functional effects and associations. RARRES1 peptide expression was 3.15-fold higher in HCC1937 cells compared to MDA-MB-468 cells, which is consistent with our qPCR analysis (2.29-fold, Figure 2). We first identified those genes which were consistently regulated between cell lines (Figure 4). Fifteen genes are either consistently up- or down-regulated in all three cell lines. We used genes up- or down-regulated in at least two of the three cell lines (as in Supplementary Figure S3) to generate a STRING [27] network (Supplementary Figure 4A). Notably, we identified SUMO2 at the center of the network. SUMO2 is downregulated in both MDA-MB-468 and HCC1937 (see Supplementary Figure S3). This supports previous findings in which RARRES1 expression was associated with SUMO2 expression in HCT116 colon cancer cells [28]. In a DAVID analysis [29,30], we identified those Gene Ontology Biological Processes enriched in at least two of the three cell lines (Supplementary Figure S4B). RARRES1 appears to affect metabolism, nucleic acid processing, and post-translational processes; however, these biological processes were not consistently identified.

To determine if the cellular localization of RARRES1 is consistent with its potential roles as suggested by the network analyses of the proteomic data, we performed confocal immunofluorescence. We examined whether RARRES1 colocalized with the endoplasmic reticulum marker, protein disulfide isomerase (PDI, Supplementary Figure S5A), with a golgi apparatus marker, giantin (Supplementary Figure S5B) or with a nuclear Topro 3 stain. Notably, RARRES1 was predominately absent from the nucleus (as seen in Supplementary Figure S5A and S5B), however; we observed a significant colocalization with PDI when compared to giantin as determined by the Costes coefficient (Supplementary Figure S5C), suggesting that RARRES1 primarily localizes to the ER. The predominant localization of RARRES1 in the ER is consistent with its function in post-translational processes and metabolism as indicated by the DAVID and STRING analyses of the mass spectrometry-identified proteins.

### RARRES1 is hypomethylated in basal-like breast cancers in the context of genome-wide hypermethylation

We then investigated the possible mechanisms for the differential expression of RARRES1 across the breast cancer subtypes. Mutations did not appear to contribute significantly to RARRES1 expression (Figure 1A), suggesting epigenetic (e.g. DNA methylation) and other transcriptional mechanisms as likely contributors.

**Figure 5:**
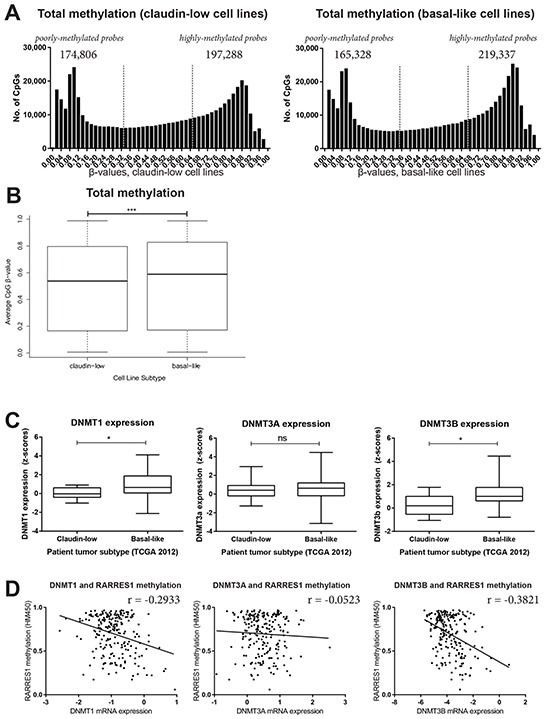
Basal-like breast cancer is more highly methylated than claudin-low breast cancer The HumanMethylation450 β-values were averaged for all claudin-low and all basal-like cell lines and are plotted **A.** as a histogram, and **B.** as a boxplot. Utilizing the 2015 TCGA breast cancer data set accessed via cBioportal [31], the expression of DNMT1, 3A and 3B was compared between basal-like and claudin-low patient tumors. Distributions were compared by **C.** a Mann-Whitney test and **D.** linear correlations.

We performed Illumina HumanMethylation450 bead chip (HM450) arrays for 26 cell lines and submitted this data to NCBI (Geo Series Accession #GSE78875; http://www.ncbi.nlm.nih.gov/geo/query/acc.cgi?acc=GSE78875. The β-values of all claudin-low cell lines (n=9) and all basal-like cell lines (n=9) were averaged and the frequency of these values were plotted (Figure 5A). The distribution of methylation in these breast cancer subtypes were significantly different, suggesting higher overall methylation in the basal-like cell lines (Figure 5B). Consistent with the overall higher methylation of the basal-like cell lines, basal-like tumors (n=81) had significantly higher levels of maintenance methyltransferase DNMT1 and *de novo* DNMT3B than the claudin-low tumors (n=8) (Figure 5C). Furthermore, the methylation of RARRES1 at cg08977270 was only weakly negatively correlated with levels of DNMT1 (r=−0.2933), or the *de novo* methyltransferases DNMT3A (r=−0.05230) and DNMT3B (r=−0.3821) (Figure 5D, N=220), in the 2015 TCGA data set [31]. Therefore, the increased expression of RARRES1 in basal-like tumors is not due to overall greater hypomethylation of basal-like cancers, and suggests an alternative hypothesis – the specific hypomethylation of RARRES1 in basal-like breast cancer.

### Methylation contributes to differential subtype-specific RARRES1 expression

**Figure 6:**
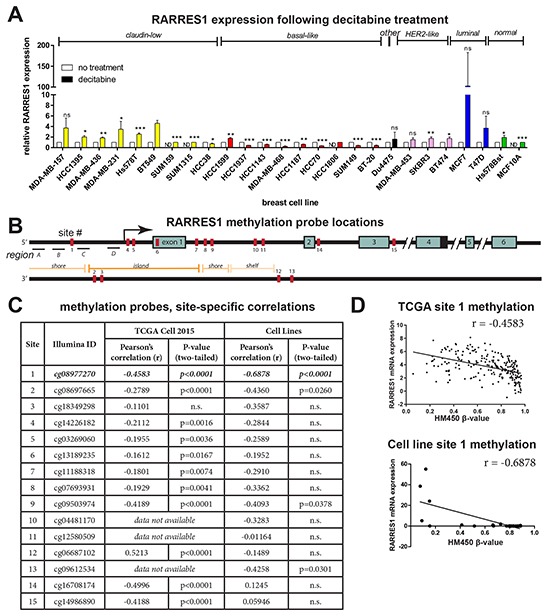
RARRES1 is hypomethylated in basal-like breast cancer **A.** The panel of24 cancerous and 2 normal breast cell lines were treated with decitabine, and RARRES1 expression was measured by qPCR; data was compared by a paired student's t-test. **B.** The locations of the HumanMethylation450 (HM450) Illumina probes and the regions used for 5-methylcytosine ChIP are plotted in relation to the RARRES1 TSS and exons. **C.** Correlations between the β-value at each HM450 site and mRNA expression of RARRES1 within the TCGA data [31] and HM450 cell line data are summarized by site. **D.** RARRES1 expression for each of 220 breast cancer samples and the 26 breast cell lines is plotted relative to the Illumina HM450 β-value at site 1. For all statistical comparisons, * p<0.05, ** p<0.01, *** p<0.001.

Having hypothesized subtype-specific hypomethylation of RARRES1, we determined if we could restore expression of RARRES1 in cell lines with low expression by treating the 26 cell lines described earlier with the demethylating agent decitabine [32]. QPCR illustrated that decitabine treatment restored RARRES1 in the luminal, Her-2-like and the majority of claudin-low cell lines, consistent with the hypermethylation of RARRES1 in these subtypes (Figure 6A). In contrast, expression of RARRES1 was decreased in basal-like cell lines, which is consistent with hypomethylation of RARRES1 in the basal-like subtype. The notable exceptions to this pattern were two basal-like cell lines HCC1599 and HCC1806, suggesting that they are hypermethylated (consistent with their low expression in Figure 2A); and the claudin-low cell line HCC38 (consistent with its high expression of RARRES1 as in Figure 2A). This pattern is consistent with the specific hypomethylation of RARRES1 in basal-like breast cancer.

Next, to determine the mechanism for the subtype-specific hypomethylation or silencing of the RARRES1 tumor suppressor, we analyzed HM450 data available from the TCGA data portal for 220 patient breast tumors. The HM450 array has 15 probes located in or near the RARRES1 gene (Figure 6B). These probes were overwhelmingly negatively correlated with RARRES1 expression (Figure 6C, Supplementary Figure S6), suggesting that DNA hypermethylation may be silencing expression of RARRES1 in the luminal, Her2-like, and claudin-low subtypes. Utilizing our HM450 array data for the 26 cell lines, we identified a strong correlation at site 1 (Figure 6C, Supplementary Figure S7), which is consistent with our findings in the TCGA 2015 data set (Figure 6D).

**Figure 7:**
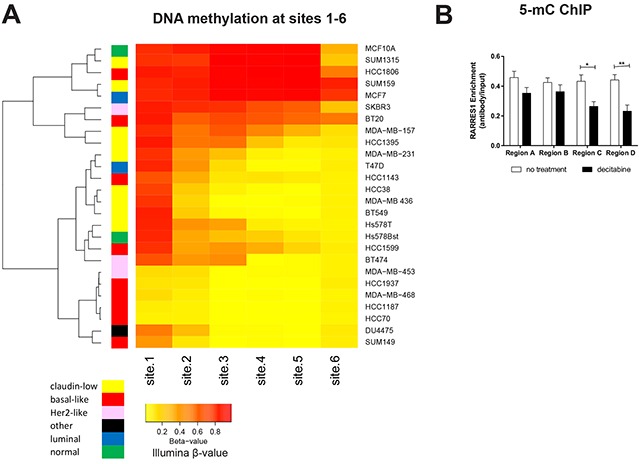
DNA methylation progressing from site 1 controls expression of RARRES1 **A.** The panel of 24 cancerous and 2 normal breast cell lines were clustered (R function, heatmap.2) based on the relative methylation at RARRES1 sites 1-6 (as quantified by HM450 β values). **B.** RARRES1 enrichment as measured by qPCR following 5-methylcytosine ChIP in Hs578T cells treated with decitabine. Each region in (C) was compared using a student's t-test (* p<0.05, ** p<0.01, *** p<0.001).

We then clustered the 26 cell lines based on methylation at sites 1 through 6, which revealed that site 1 is the primary region initiating progressive DNA methylation into the gene body and illustrates the clustering of basal-like breast cancers by specific hypomethylation of the region (Figure 7A). In validation of the importance of methylation of the promoter region of RARRES1, we performed 5-methylcytosine (5-mC) ChIP on RARRES1-silenced Hs578T cells using 4 locations ranging from ~1000bp upstream of the transcription start site (TSS) to within 100bp of the TSS (as described in Peng, 2012 [17], indicated in Figure 6B as A-D). We observed a decrease in 5-mC following decitabine treatment which was most pronounced in Region C and D (Figure 7B), which is located nearest to site 1. This is consistent with our identification of a region containing site 1 as the region most important for initiating epigenetic silencing via DNA methylation.

### ALDH1A3 is a secondary factor that determines RARRES1 expression in TNBC

Although our data thus far suggest the importance of DNA methylation as a major factor dictating the expression of RARRES1 in breast cancer subtypes, our previous work and the presence of retinoic acid response elements (RAREs) in the gene suggest that the transcription mediator RA also plays a role in subtype-specific expression of RARRES1. RA is generated physiologically by the retinaldehyde dehydrogenases ALDH1A1, ALDH1A2, and ALDH1A3. Once synthesized, RA binds to the retinoic acid and retinoid X receptors (RARs and RXRs) located at genomic RAREs [33]. This catalyzes the release of co-repressors and recruits co-activators to induce transcription of RARE-containing genes, such as RARRES1 [34].

**Figure 8:**
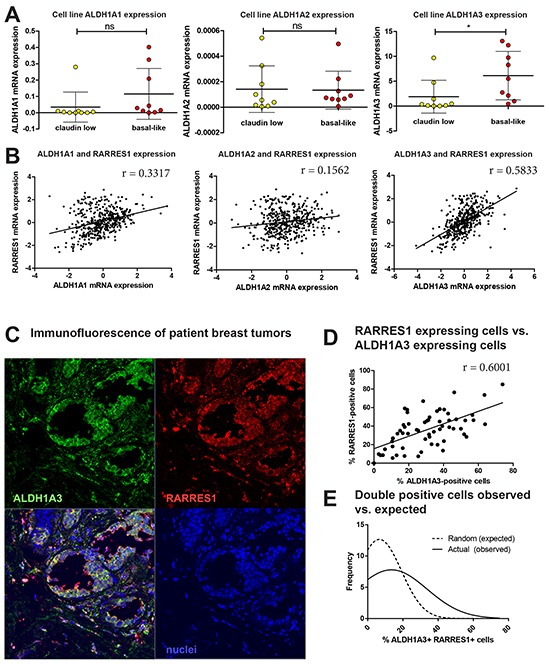
ALDH1A3 expression correlates and colocalizes with RARRES1 expression in patient tumors **A.** Expression of ALDH1A1, ALDH1A2 and ALDH1A3 were compared among all claudin-low and basal-like cell lines. **B.** Using the TCGA data via cBioportal [31], mRNA expression of ALDH1A1, ALDH1A3, and ALDH1A3 were correlated with RARRES1 via a linear correlation. **C.** Representative image of 62 formalin fixed primary breast cancer patient tumor samples stained for ALDH1A3 and RARRES1 protein expression by immunofluorescence. **D.** Quantification of the percentage of RARRES1-positive cells was correlated as a function of the percentage of ALDH1A3-positive cells in 62 individual patient tumor samples as detected by immunofluorescence (linear correlation). **E.** The percentage of double-positive cells in the immunofluorescent images were determined as a function of percentage of cells that were positive for RARRES1 and ALDH1A3 protein expression in and graphed as a Gaussian distribution of random (expected) double-positive cells, and compared to a Gaussian distribution of actual (observed) double-positive cells using an extra sum-of-squares F test.

We first identified whether expression of the RARs and RXRs (α, β, and γ) correlated with expression of RARRES1 in the 2015 TCGA data set [31]. We did not observe any relevant correlation between RARRES1 expression and RAR/RXR expression (Supplementary Figure S9), suggesting that expression of these nuclear receptors was not dictating expression of RARRES1 in breast cancer. We continued upstream in RA signaling and investigated the possible connection between RARRES1 and the RA-producing ALDH1A1, ALDH1A2, and ALDH1A3. In all 26 cell lines except SUM159 and BT474, ALDH1A3 was the most highly expressed isoform (Supplementary Figure S9). Similar to our findings with RARRES1, we observed significantly higher expression of ALDH1A3 in the basal-like cell lines when compared to the claudin-low cell lines (p<0.01, Figure 8A). This suggests that if RA-producing enzymes are playing a role in RARRES1 subtype-specific regulation, ALDH1A3 is most likely the main contributor.

Next, we obtained data from TCGA [18] that demonstrates weak correlations between RARRES1 and ALDH1A1, and ALDH1A2, but a moderately strong and significant correlation between RARRES1 and ALDH1A3 (N=460, p<0.001) (Figure 8B). To investigate if this correlation exists beyond the mRNA level, we assessed RARRES1 and ALDH1A3 protein expression in 62 primary breast cancer tumors by immunofluorescence and found a significant correlation between the percentage of cells expressing ALDH1A3 and RARRES1 (Figure 7C and 7D). The expected random probabilities and the actual observed percentage of cells positive for both RARRES1 and ALDH1A3 were plotted as a histogram and fit with a Gaussian distribution (Supplementary Figure S10A). The Gaussian distributions were compared (Figure 7E) and the mean actual percentage of double-positive cells (20.78%) is significantly higher than that expected due to random probability (12.83%, p<0.05). These correlations between ALDH1A3 and RARRES1 suggests that expression of RARRES1 in breast cancer is not only controlled by methylation in the promoter region, but also by ALDH1A3 via its production of RA. Importantly, this assumption was corroborated by knockdown of ALDH1A3 in MDA-MB-468 cells [15], which also reduced protein expression of RARRES1 (Supplementary Figure S10B).

### DNA methylation and ALDH1A3/RA co-regulate expression of RARRES1

**Figure 9:**
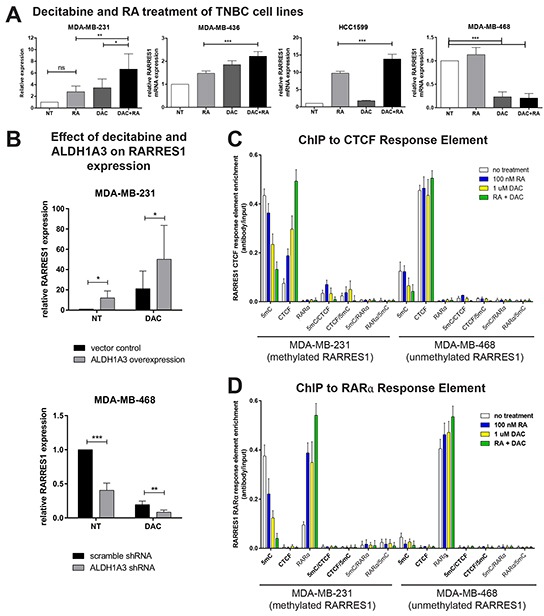
RARRES1 expression is influenced by DNA methylation and retinoic acid signaling **A.** The effect of decitabine (DAC) and retinoic acid (RA), alone or combined on RARRES1 expression was determined by qPCR in methylated cell lines (MDA-MB-231, MDA-MB-436, HCC1599), and unmethylated MDA-MB-468 cells. Treatments were compared using a repeated-measures ANOVA. **B.** The effect of ALDH1A3 overexpression in MDA-MB-231 cells (have low levels of intrinsic ALDH1A3) and ALDH1A3 knockdown in MDA-MB-468 cells (have high levels of intrinsic ALDH1A3) on RARRES1 expression was determined by qPCR. Decitabine-treated values were compared to no-treatment values using a paired student's t-test. **C.** To interrogate the RARRES1 promoter, ChIP and double ChIP assays on were performed on MDA-MB-231 cells (have methylated RARRES1 promoter and low levels of ALDH1A3, which produces RA) and MDA-MB-468 cells (have unmethylated RARRES1 promoter and intrinsic high ALDH1A3) that were either treated with decitabine (DAC), retinoic acid (RA) or both. The assays were performed using antibodies against 5-mC, RARα and CTCF, as well as the control normal rabbit IgG, alone or in combination for the double ChIP assays. In the double ChIP assays only DNA sequences that bind both proteins concurrently are detected by this assay.

Having established that both DNA hypomethylation and high expression of RA-producing ALDH1A3 are factors in the subtype-specific expression of RARRES1, we next assessed how these factors control RARRES1 expression together. We examined the mRNA expression of RARRES1 following treatment with RA/changes in ALDH1A3 expression, decitabine, or a combination of both. Demethylation of RARRES1 with decitabine allows or enhances RA-dependent transcription of the gene (Figure 9A). We observed consistent results when ALDH1A3 was overexpressed in MDA-MB-231 or knocked down in MDA-MB-468 (Figure 9B). This suggests that while DNA methylation is key in controlling the expression of RARRES1, physiological RA produced by ALDH1A3 is also a determinant for RARRES1 expression.

We then used ChIP to validate RA as an important secondary determinant in RARRES1 expression. Treatment of RARRES1-methylated MDA-MB-231 cells and RARRES1-unmethylated MDA-MB-468 cells with both RA and decitabine is required for maximal demethylation of the RARRES1 gene (illustrated by decreased binding to the 5-mC antibody, Figure 9C and 9D). Additionally, RA and decitabine are both required for maximal binding of both CTCF (a multipurpose DNA binding protein, Figure 9C), and RARα (the nuclear receptor of RA, Figure 9D) to their respective response elements in RARRES1. While CTCF can have multiple functions including transcriptional activation and repression [35], it appears to activate transcription at an unmethylated RARRES1 promoter [17].

The double-ChIP with 5-mC and CTCF (Figure 7C) or RARα (Figure 7D) demonstrates that CTCF and RARα do not bind to methylated DNA. This supports our finding that demethylation is required for maximal induction of RARRES1 transcription, and corroborates the wide range of RARRES1 expression values identified between the RARRES1-methylated claudin-low cell lines and the RARRES1-unmethylated basal-like cell lines.

## DISCUSSION

RARRES1 was first described as a novel retinoid response gene in skin raft cultures [36]. RARRES1 is a commonly silenced hypermethylated locus in many cancer types including prostate cancer [25], hepatocellular carcinoma [37], and breast cancer [17]. Although generally described as a putative tumor suppressor gene, a recent report indicated a pro-tumorigenic role for RARRES1 in a rare form of breast cancer, inflammatory breast cancer [16]. In contrast, in this study we identified RARRES1 as a tumor suppressor in TNBCs, and highly expressed specifically within the basal-like subtype. We determined that subtype-specific expression of this tumor suppressor is due to both its specific hypomethylation, and ALDH1A3 expression within basal-like breast cancers, which provides its necessary transcription induction molecule, RA, for nuclear hormone receptor RARα. Our characterization of the RARRES1 gene offers an example of a subtype-specific tumor suppressor that may be useful as a biomarker in subtype-specific therapies.

The heterogeneity of breast cancer complicates therapeutic decision making and affects patient outcomes. Recent research has focused on identifying gene expression profiles, mutational maps, and methylation profiles to identify different subtypes of breast cancer [18,31,38]. These have revealed that the genes expressed in these different subtypes are important in determining the response of patients to anti-cancer therapies. Importantly, the specific expression and hypomethylation of RARRES1 in basal-like breast cancer adds RARRES1 to a list of genes which are differentially regulated and expressed in breast cancer subtypes [39, 40]. These genes may correlate with, or be causative factors in, the varying responses of different subtypes to various chemotherapy regimens.

In particular, RARRES1 is an RA-inducible tumor suppressor gene. This is in direct contrast with the vast majority of tumor suppressors, which are currently considered as undruggable except by complex synthetic or conditional lethality models [41]. While RA has achieved limited clinical success in breast cancer, an increasing body of work suggests that RA affects key processes important for the progression and metastasis of breast cancer in a context-specific manner. For example, RA signaling exhibits either cooperative or antagonistic interplay with estrogen signaling [42,43]; RA can promote either a pro-apoptotic or a pro-survival response [44]; or RA can promote or suppress TNBC tumor growth [15]. We recently hypothesized that differential methylation of tumor-suppressive and pro-growth genes in breast cancer may affect the response of breast cancers to RA therapy [15]. RARRES1 is one example of a gene that fits this paradigm and may suggest that a specific subtype of breast cancer (i.e. basal-like breast cancers) could be treated with RA.

## MATERIALS AND METHODS

### Ethics statement

Animal investigations detailed in this manuscript have been conducted in accordance with the ethical standards and according to the Declaration of Helsinki and according to national and international guidelines. All experiments were conducted in accordance with the Canadian Council on Animal Care standards and a protocol approved by Dalhousie University Committee on Laboratory Animals (#13-010). Patient samples were collected and analyzed in accordance with protocol #1007106, approved by the IWK Health Centre Research Ethics Board.

### Cell lines, vectors, and reagents

Cell lines were obtained from the American Type Culture Collection (ATCC) and cultured as described in Supplementary Table 1. RARRES1 shRNA knockdown clones were generated as previously described [15,45], using the pGipZ lentiviral vector (shRNA 1: V3LHS_398249; shRNA 2: V3LHS_398251; Dharmacon). Western blotting was used to verify RARRES1 expression (R&D anti-RARRES1, cat#AF4255, 1/300).

For 5-aza-2′-deoxycitidine (DAC) treatment, 1 μM DAC (Sigma) was added for 72 hours and replaced every 24 h. When used in combination with all-trans retinoic acid (RA), 100 nM RA (Sigma) was added for the last 18 h.

### Quantitative PCR

QPCR was performed on cDNA generated from extracted RNA as previously described using gene-specific primers (Supplementary Table 2). Standard curves for each primer set were generated, and primer efficiencies were incorporated into the CFX Manager software (Bio-Rad). mRNA expression of all samples was calculated relative to two reference genes [glyceraldehyde 3-phosphate dehydrogenase (GAPDH) and β-2-microglobulin (B2M) for analyses within cell lines; ADP-ribosylation factor 1 (ARF1) and pumilio homolog 1 (PUM1) for analyses between cell lines].

### Cell proliferation analyses

Cells were seeded in 6 well plates at 2.5 × 10^4^ cells/well. Cells were counted 24 h after seeding and 144 h after seeding. Data was normalized to the number of cells at 24 h, and proliferation was determined relative to the scramble shRNA.

### Tumor tissue histological analysis by immunofluorescence microscopy

Formalin fixed and paraffin embedded breast cancer patient tumor core biopsy tissue were taken post-surgery from consenting patients who were diagnosed with breast cancer at the Queen Elizabeth II Health Sciences Centre (QEII HSC) in Halifax, NS, Canada between 2007 and 2014. Standard pathological assessments of patient tumors were performed by staff pathologists at the QEII HSC (Supplementary Table S2). Sequential sections were stained with anti-ALDH1A3 (Abgent) and anti-RARRES1 (Abcam) and species-specific secondary antibodies, conjugated to either Cy2 or Cy3 (Jackson Immunoresearch) and nuclear stain To-Pro-3 (Invitrogen). Images were captured with a Zeiss LSM 510 laser scanning confocal microscope and quantified as previously described [45].

### Tumor xenograft studies

Eight-to-ten week-old NOD/SCID mice were injected orthotopically in the mammary fat pad with 2 × 10^6^ MDA-MB-231 or MDA-MB-468 cells (vector control and RARRES1 shRNA clones). Injected cells were mixed 1:1 with high-concentration Matrigel (BD Biosciences). Primary tumor growth was quantified (length × width × depth × 0.5) and modeled using a quadratic non-linear regression, and compared with an extra sum-of-squares F test.

### Methylation profiling

DNA was collected from untreated and DAC-treated cells using the PureLink DNA kit (Invitrogen). Methylation analyses using the HM450 array (Illumina) was performed by the Centre for Applied Genomics at the Hospital for Sick Children (Toronto, Ontario, Canada) including bisulfite conversion, hybridization, background subtraction, and normalization (Geo Series Accession #GSE78875; http://www.ncbi.nlm.nih.gov/geo/query/acc.cgi?acc=GSE78875). β-values for Illumina probes near RARRES1 were extracted from the data, and locations determined relative to the protein-coding regions.

### cBioportal data analysis

Data from TCGA [18,31] were analyzed with cBioportal [19,20] or extracted from the TCGA Data Portal as indicated.

### Chromatin immunoprecipitation

ChIP assays [46] were performed following the ChIP assay kit protocol (cat#06-599, Upstate Biotechnology) as previously described [47] using antibodies against 5-mC (cat#BI-MECY-0500, AnaSpec, Inc.), RARα (cat#ab41934, abcam), CTCF (cat#07-729, Millipore) as well as the control normal rabbit IgG (cat#sc-2027, Santa Cruz Biotechnology). After dissociating the DNA-protein complexes, pulled-down DNA along with the input DNA (devoid of antibody) were subject to qPCR analysis with primers to interrogate the RARRES1 promoter (Supplementary Table 2). Results are expressed as the amount of DNA detected in the immunoprecipitated fraction minus the amount of DNA detected in the nonimmune IgG (negative control) fraction normalized to the input DNA. For sequential ChIP (ChIP–reChIP) experiments, the protein bound to the beads with the first antibody was incubated (30 min, 37°C) twice with DTT (20 mM) and the combined elutes were suspended in ChIP dilution buffer, which was then immunoprecipitated (14 h, 4°C) with the second antibody.

### Protein analyses and mass spectrometry

Western blotting was used to detect changes in AXL expression (R&D, cat#AF154, 1/300) and detyrosinated tubulin (tubulinEE, AbD serotec, cat#obt1660, 1/1000; α-tubulin, Sigma-Aldrich, cat#T9026-DM1A, 1/1000).

For mass spectrometry, preparation of lysates, protein digestion, and peptide labelling were performed as previously described [48]. Labeled and mixed peptides were fractionated into 12 fractions using basic pH reverse-phase HPLC on a monolithic (100 mm × 4.6 mm) reversed phase column (Phenomenex). Fractions were analyzed using 3 hr gradients from 0-40% acetonitrile (0.1% formic acid) on an Orbitrap Velos Pro mass spectrometer (Thermo-Fisher) using MS3 acquisition as described [49]. All MS data were processed as previously described [50].

### Statistical analyses

All statistical analyses were calculated in GraphPad Prism 6 unless indicated otherwise. Paired t-tests were used to compare two treatments, one-way ANOVA was used for multiple treatments. Unpaired t-tests were used to compare groups of cell lines or mice. For all comparisons, * p<-0.05, ** p<0.01, *** p<0.001.

## SUPPLEMENTARY MATERIALS AND METHODS






