# Supplementary material for: Breast cancer subtype dictates DNA methylation and ALDH1A3-mediated expression of tumor suppressor RARRES1
Source: Oncotarget. 2016 Jun 6;7(28):44096–112. doi: 10.18632/oncotarget.9858 (PMC5190082; doi:10.18632/oncotarget.9858)
Supplement: Supplementary file 1 [file oncotarget-07-44096-s001.pdf]

Breast cancer subtype dictates DNA methylation and ALDH1A3-mediated expression of tumor suppressor RARRES1

SUPPLEMENTARY MATERIALS AND METHODS

Immunohistochemical staining and microscopy

MDA-MB-468, and SUM149 cells were seeded at approximately 35% confluency onto poly-L-lysine coated coverslips, in 12 well plates. After 24 h, the coverslips fixed in 3% paraformaldehyde, and permeabilized with 0.1% Triton-X-100 in PBS. Following permeabilization the cells were blocked with 1% bovine serum albumin (BSA) and then incubated overnight with 1/500 dilution of primary antibodies: monoclonal mouse anti-RARRES1 (Abcam, ab92884), monoclonal rabbit anti-PDI (Covance, PRB-114P), and polyclonal rabbit anti-Giantin (Abcam, ab31811). Coverslips were stained with species-specific Alexafluor 488nm or Cy3 conjugated secondary antibodies (Jackson Immunoresearch) and ToPro 3 (ThermoFisher). Mounted coverslips were imaged with the Zeiss 510 Laser Scanning Confocal Microscope, using the Zen 2012 software. Images of MDA-MB-468 cells were captured under a 40x oil immersion objective lens, with a 10x optical lens, for a total magnification of 400x. Images of SUM149 cells were captured under a 63x oil immersion objective lens, with a 10x optical lens, for a total magnification of 630x. Quantitative image analysis was done using ImageJ (Fiji). The Colocalization Threshold plugin was used to determine the tM1 coefficient (a value from 0 to 1) using the Costes' method (red channel overlapping with green) (Costes, 2004). Each image was analysed using the Colocalization Threshold plugin to determine the Costes' coefficient. The 5 technical replicates per slide and three experimental replicates were averaged. Statistical analysis of the tM1 values was performed using a paired t-test in GraphPad Prism 6.

ALDH1A3 knockdown

ALDH1A3 knockdown and overexpression cells were generated previously and validated by western blotting as described previously[42]. Cells were selected with 1.5 μg/mL puromycin and maintained in 0.25 μg/mL puromycin.

REFERENCES

1. Prat A, Karginova O, Parker JS, Fan C, He X, Bixby L, Harrell JC, Roman E, Adamo B, Troester M, Perou CM. Characterization of cell lines derived from breast cancers and normal mammary tissues for the study of the intrinsic molecular subtypes. Breast Cancer Res Treat. 2013:142:237–55.
2. Prat A, Parker JS, Karginova O, Fan C, Livasy C, Herschkowitz JI, He X, Perou CM. Phenotypic and molecular characterization of the claudin-low intrinsic subtype of breast cancer. Breast Cancer Res. 2010:12:R68.
3. Riaz M, van Jaarsveld MTM, Hollestelle A, Prager-van der Smissen WJC, Heine AAJ, Boersma AWM, Liu J, Helmjr J, Ozturk B, Smid M, Wiemer EA, Foekens JA, Martens JWM. miRNA expression profiling of 51 human breast cancer cell lines reveals subtype and driver mutation-specific miRNAs. Breast Cancer Res. 2013:15:R33.
4. Hackett AJ, Smith HS, Springer EL, Owens RB, Nelson-Rees WA, Riggs JL, Gardner MB. Two syngeneic cell lines from human breast tissue: the aneuploid mammary epithelial (Hs578T) and the diploid myoepithelial (Hs578Bst) cell lines. J Natl Cancer Inst. 1977:58:1795–806.
5. Soule HD, Maloney TM, Wolman SR, Peterson WD Jr, Brenz R, McGrath CM, Russo J, Pauley RJ, Jones RF, Brooks SC. Isolation and characterization of a spontaneously immortalized human breast epithelial cell line, MCF-10. Cancer Res. 1990:50:6075–86.
6. Marcato P, Dean CA, Liu R-Z, Coyle KM, Bydoun M, Wallace M, Clements D, Turner C, Mathenge EG, Gujar SA, Giacomantonio CA, Mackey JR, Godbout R, et al. Aldehyde dehydrogenase 1A3 influences breast cancer progression via differential retinoic acid signaling. Mol Oncol. 2015;9:17–31.
7. Marcato P, Dean CA, Pan D, Araslanova R, Gillis M, Joshi M, Helyer L, Pan L, Leidal A, Gujar S, Giacomantonio CA, Lee PWK. Aldehyde dehydrogenase activity of breast cancer stem cells is primarily due to isoform ALDH1A3 and its expression is predictive of metastasis. Stem Cells Dayt Ohio. 2011;29:32–45.
8. Peng Z, Shen R, Li Y-W, Teng K-Y, Shapiro CL, Lin H-JL. Epigenetic repression of RARRES1 is mediated by methylation of a proximal promoter and a loss of CTCF binding. PloS One. 2012;7:e36891.

SUPPLEMENTARY FIGURES AND TABLES

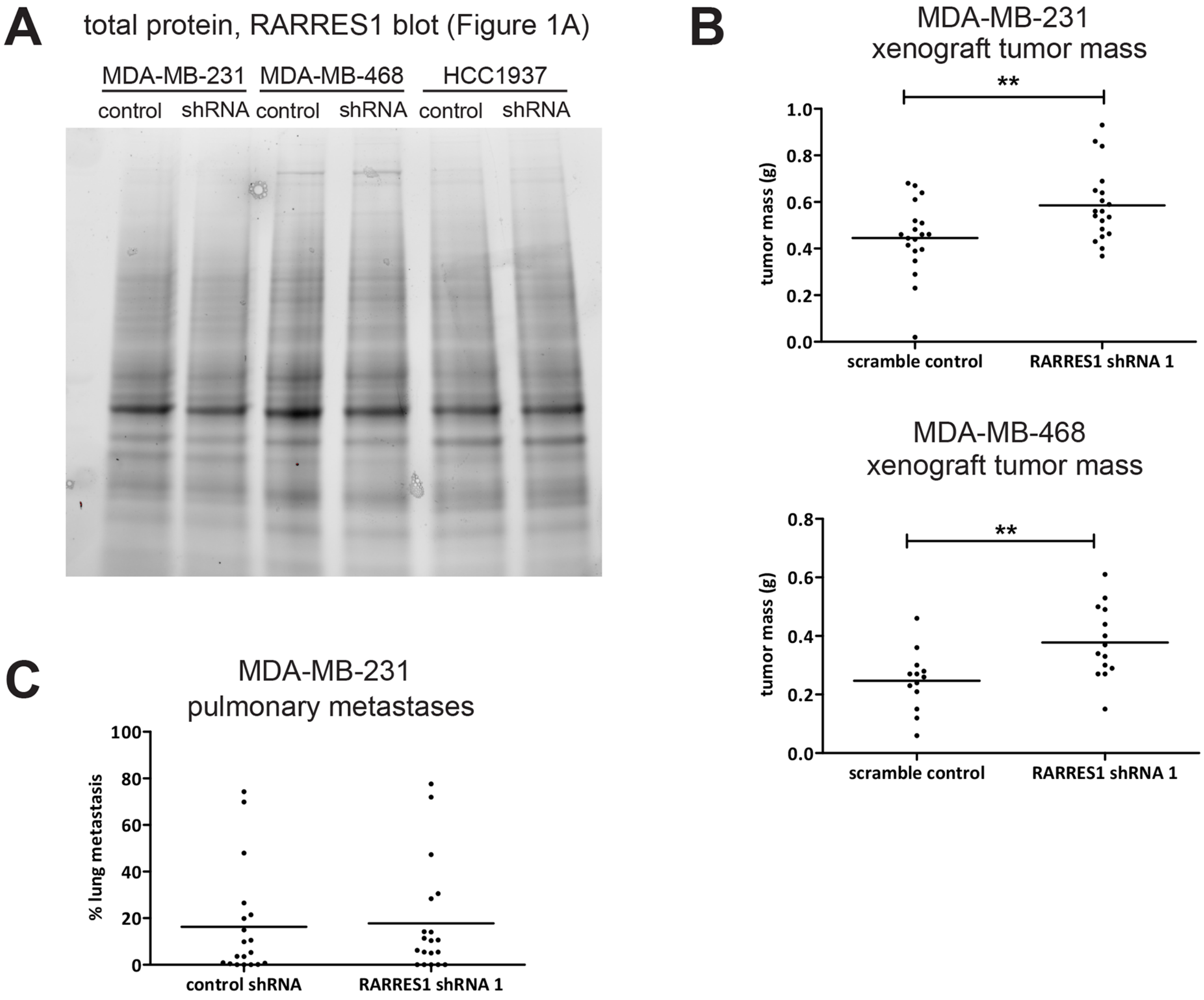

Supplementary Figure S1: RARRES1 knockdown increases tumor mass but not pulmonary metastases. A. Xenografts isolated from MDA-MB-231 and MDA-MB-468 tumor-bearing mice were massed and plotted. **B.** Lungs were harvested from tumor-bearing mice, formalin-fixed and paraffin-embedded and 5µM thin sections generated for metastasis visualization by haematoxylin and eosin (H&E) staining. Metastasis quantification of each lung were performed blinded using a standardized grid imposed on Axiocam HRC Color images captured of at least two random H&E stained thin sections/tissue. Percentage of metastatic lung tissue was calculated by dividing metastatic grid cell counts by the entire tissue grid cell counts.

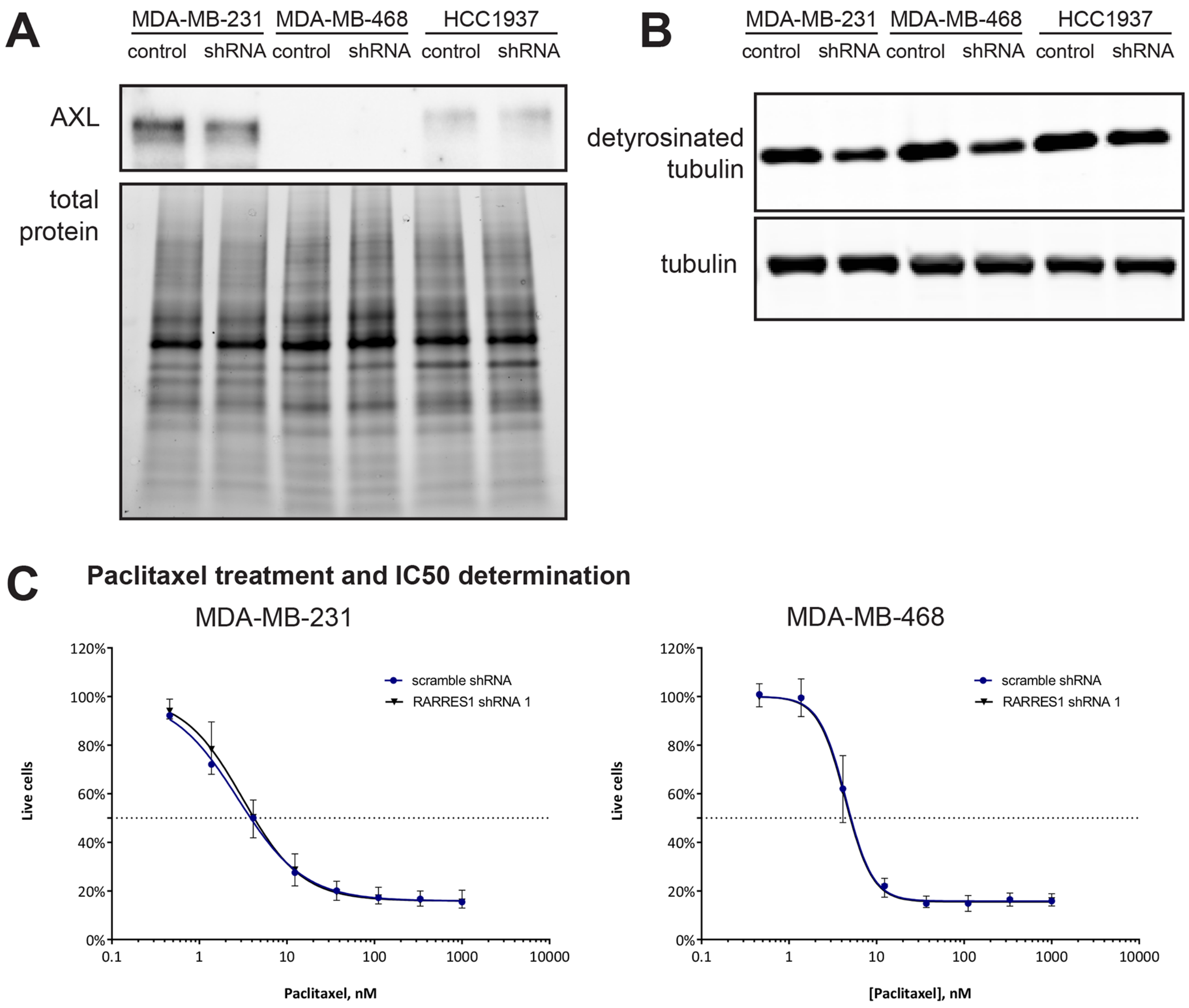

Supplementary Figure S2: The tumor suppressive function of RARRES1 is not related to AXL expression or tubulin tyrosination. RARRES1 knockdown cells in MDA-MB-231, MDA-MB-468, and HCC1937 cells were examined for **A.** AXL expression; and **B.** detyrosinated tubulin via western blot. **C.** MDA-MB-231 and MDA-MB-468 scramble control and RARRES shRNA-bearing cells were treated with varying amounts of paclitaxel and the IC50 was determined.

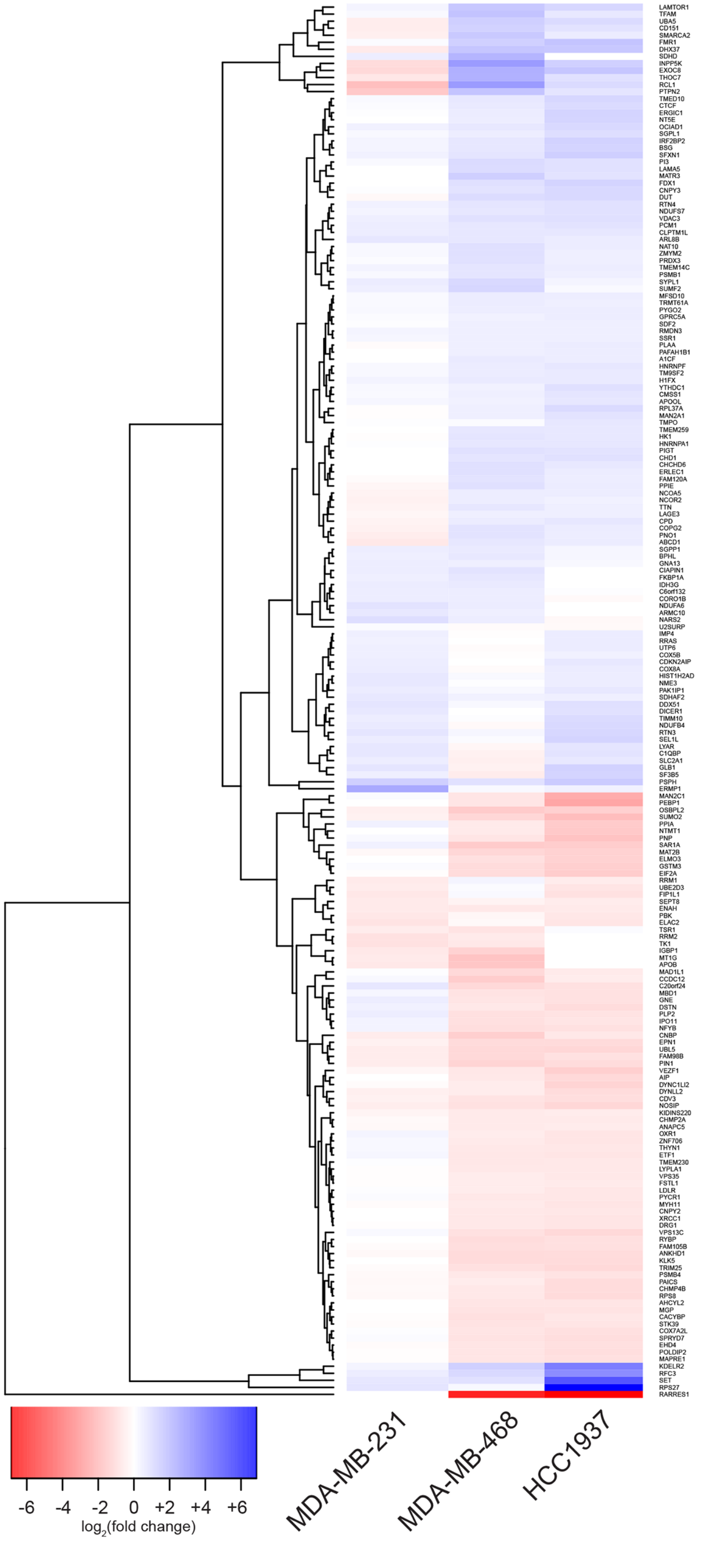

Supplementary Figure S3: Knockdown of RARRES1 affects a complex network of proteins represented in numerous pathways. A. Proteins upregulated (log_2(fold change) > 0.379) or downregulated (log_2(fold change) < -0.515), in at least two of three cell lines following RARRES1 knockdown, were input into STRING . Proteins which were not connected to the displayed central network by evidence-based interactions were removed. **B.** All proteins upregulated or downregulated in each cell line were input into DAVID. Any Gene Ontology Biological Processes (GO BP) which were enriched (Benjamini adjusted p-value < 0.05) are displayed with the corresponding number of hits represented.

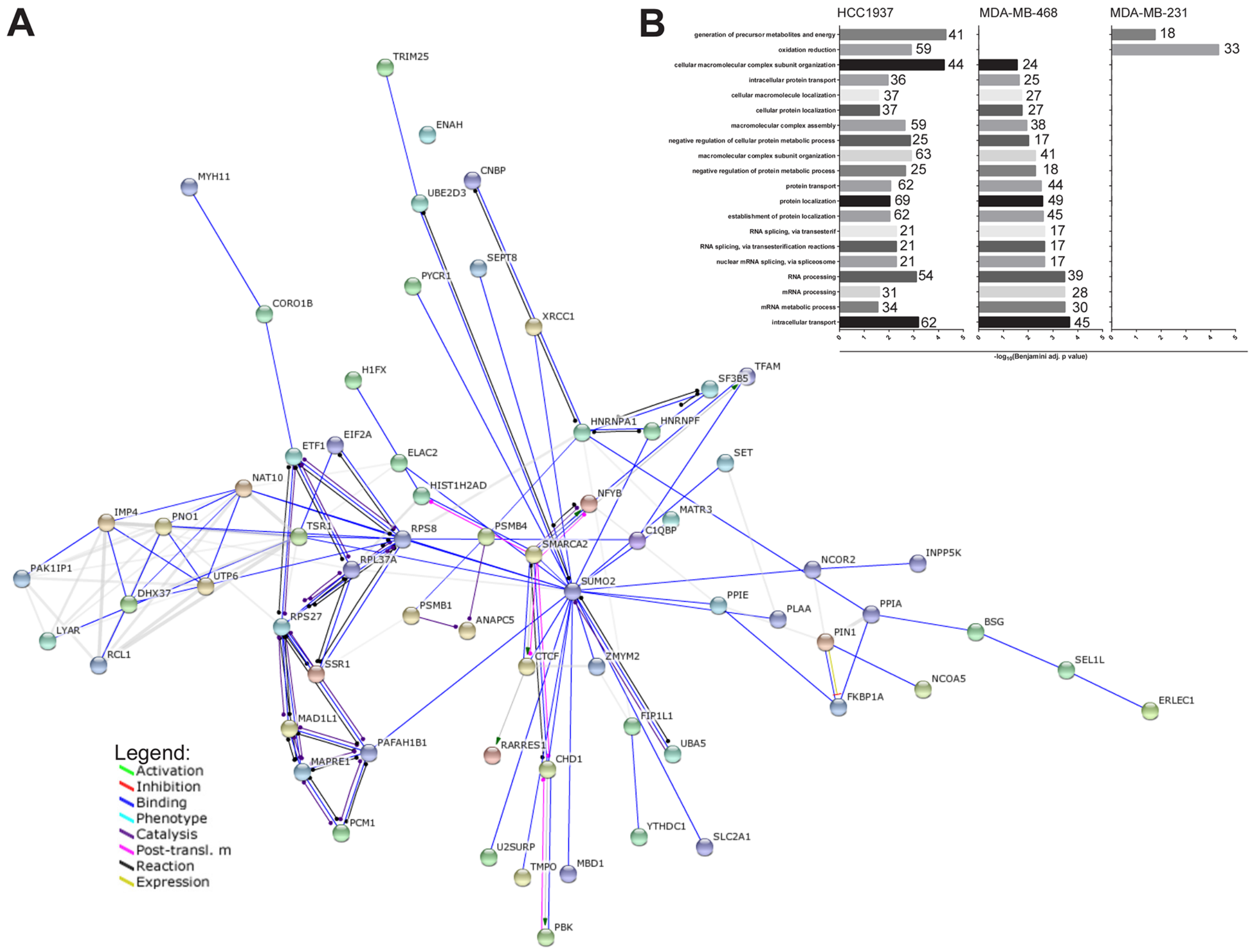

Supplementary Figure S4: Knockdown of RARRES1 affecting expression of proteins in two of the three cell lines. All proteins upregulated or downregulated in at least two cell lines (as described in Figure 4) were clustered using the heatmap.2 function (gplots, R).

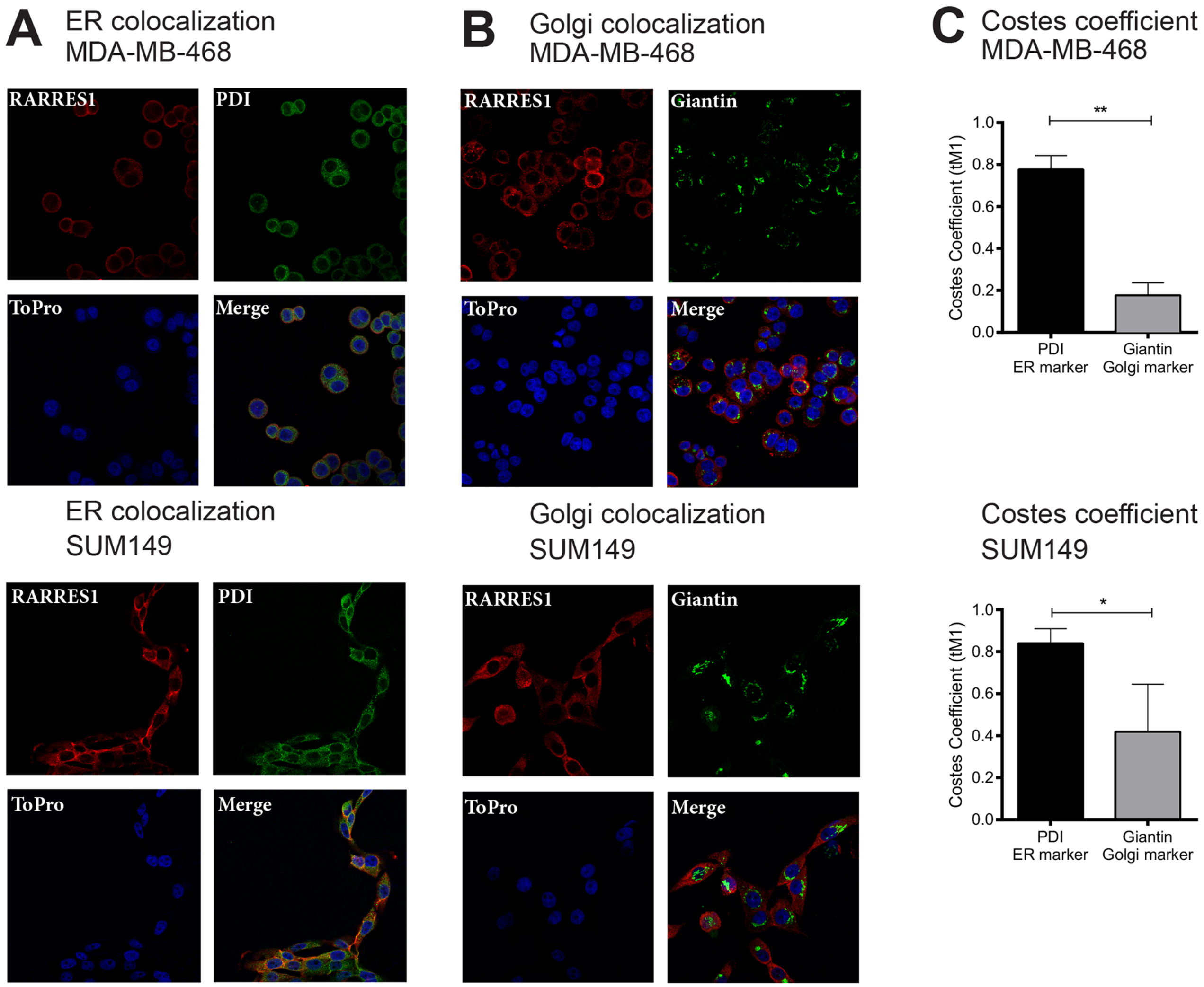

Supplementary Figure S5: RARRES1 is localized to the endoplasmic reticulum in MDA-MB-468 and SUM149. Representative images from immunofluorescence and confocal microscopy of **A.** RARRES1 and ER colocalization using PDI; and **B.** RARRES1 and Golgi apparatus colocalization using giantin in both MDA-MB-468 and SUM149. **C.** The Costes coefficient (a measure of colocalization) was calculated in both cell lines for RARRES1 with PDI and with giantin. These were compared by paired t-tests.

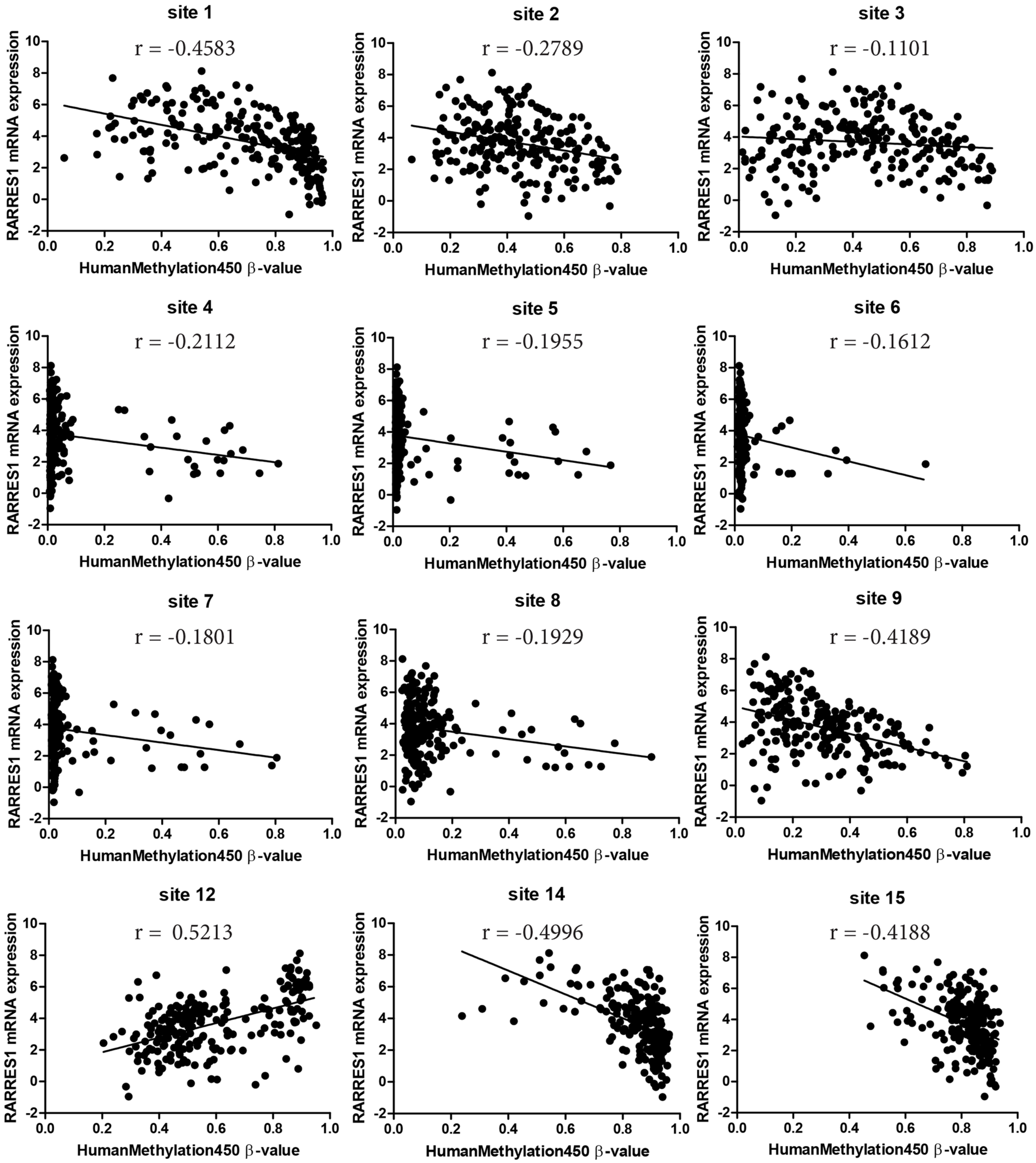

Supplementary Figure S6: RARRES1 methylation in patient samples negatively correlates with mRNA expression. Data from the TCGA Data Portal (Cell, 2015) using HM450 arrays was used to correlate mRNA expression of RARRES1 in patient tumors with the β-value at each probe location. Values on each graph represent linear correlations.

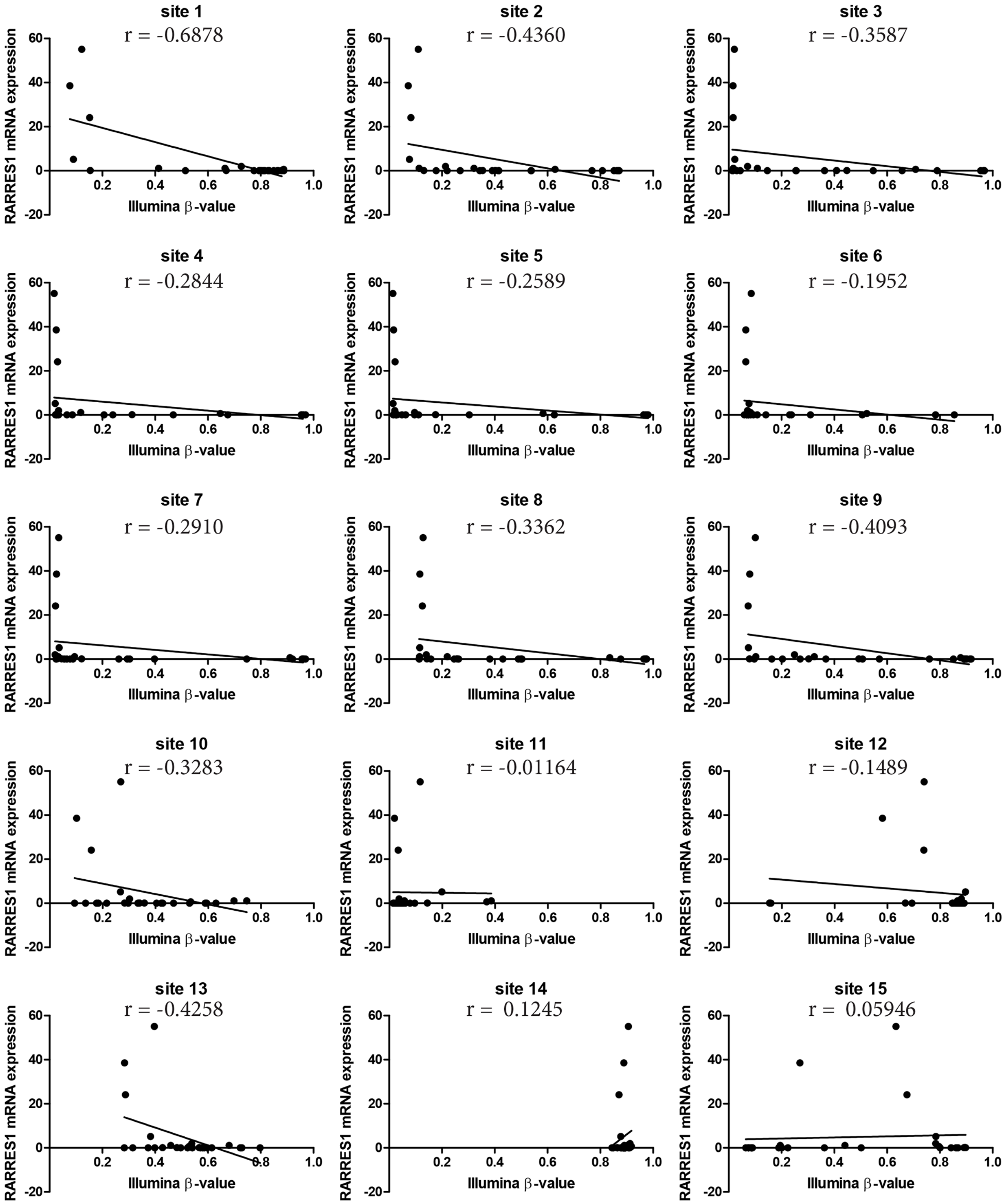

Supplementary Figure S7: RARRES1 methylation in breast cancer cell lines negatively correlates with mRNA expression. Using our own data from Illumina HM450 arrays and qPCR, mRNA expression of RARRES1 in 26 cell lines was correlated with the β-value at each probe location. Values on each graph represent linear correlations.

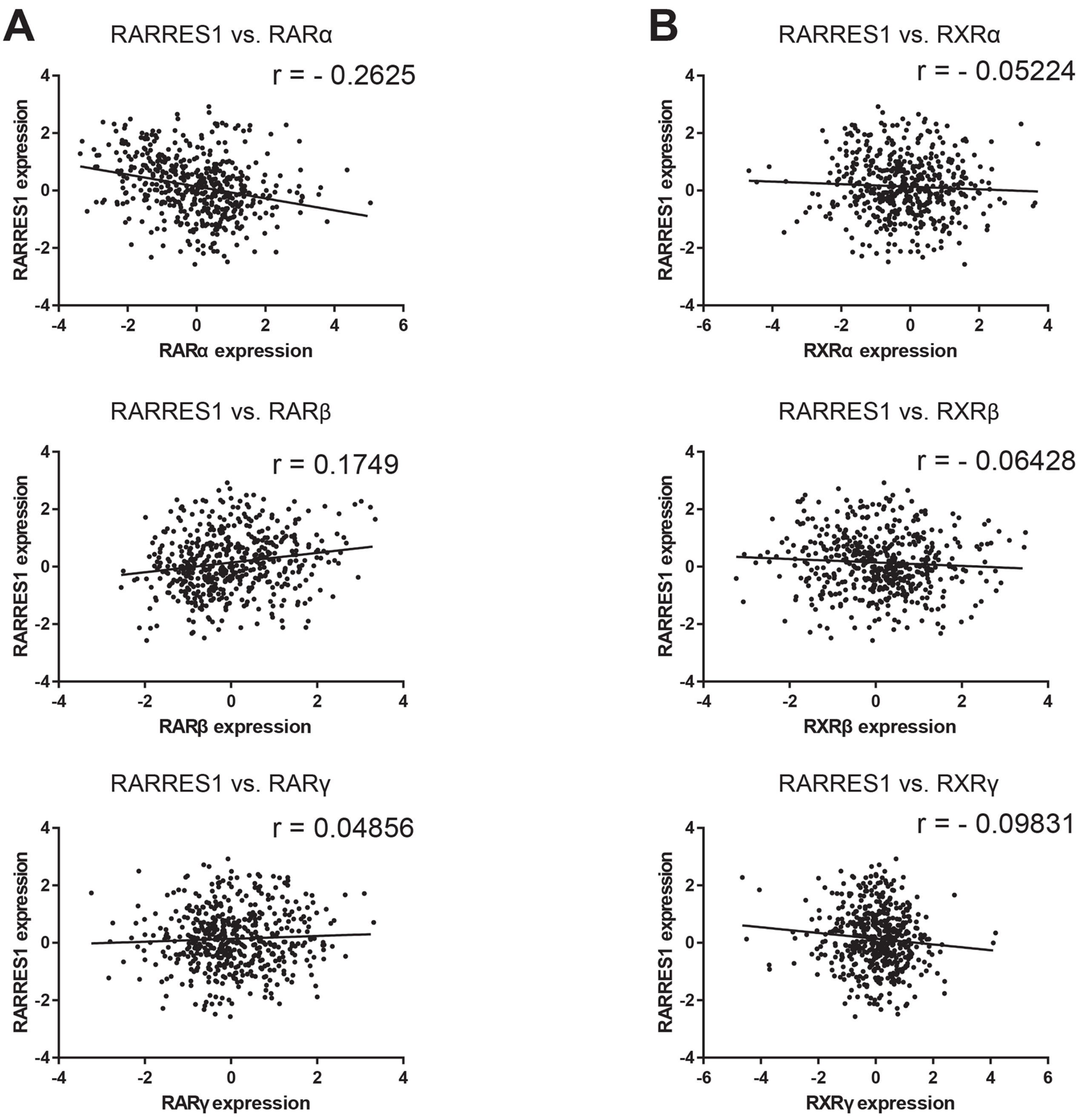

Supplementary Figure S8: RARRES1 expression does not strongly correlate with expression of RAR and RXR isoforms. Expression of RARRES1 mRNA in the TCGA (Cell, 2015) data set was correlated with expression of **A.** RARα, RARβ, and RARγ; and **B.** RXRα, RXRβ, and RXRγ.

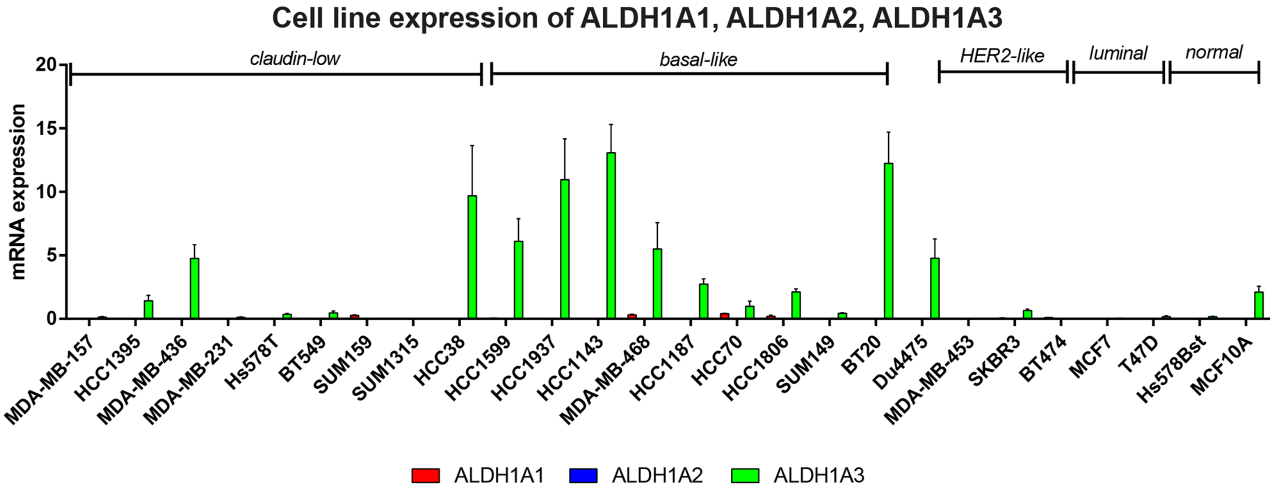

Supplementary Figure S9: ALDH1A3 is the most highly expressed ALDH1a isoform in TNBC cell lines. Expression of ALDH1A1, ALDH1A2, and ALDH1A3 mRNA in 26 cell lines was determined by qPCR.

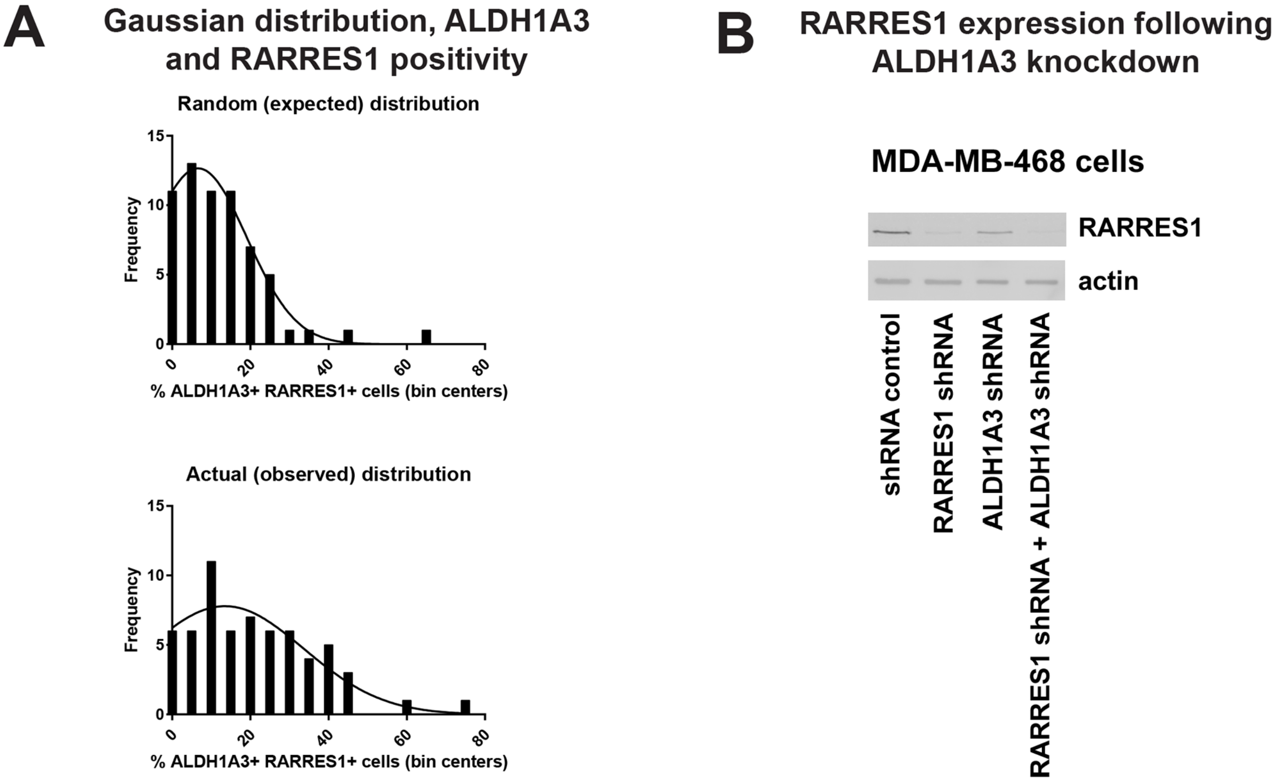

Supplementary Figure 10: ALDH1A3 correlates with and contributes to RARRES1 expression. A. Using the percent-RARRES1-positive cells and the percent-ALDH1A3-positive cells (as shown in Figure 7D), a random expected distribution of double-positive cells was determined, plotted as a histogram, and fit with a Gaussian distribution. The actual observed distribution of double-positive cells was plotted as a histogram and fit with a Gaussian distribution. These distributions are combined and compared in Figure 7E. **B.** RARRES1 expression was observed via western blotting following shRNA knockdown of ALDH1A3 and/or RARRES1 in MDA-MB-468 cells.

Supplementary File S1: Data from mass spectrometry, gene lists used for analysis.

See Supplementary File 1

Supplementary Table S1: Cell line subtypes and culture specifications

See Supplementary File 2

Supplementary Table S2: Primers utilized for qRT-PCR and ChIP

Gene		Primer	Reference
RARRES1	F	ACGGCTCATCGAGAAAAAGA	[6]
	R	GAAAGCCAAATCCCAGATGA	
ALDH1A1	F	TGTTAGCTGATGCCGACTTG	[7]
	R	TTCTTAGCCCGCTCAACACT	
ALDH1A2	F	CTGGCAATAGTTCGGCTCTC	[7]
	R	TGATCCTGCAAACACTGCTC	
ALDH1A3	F	TCTCGACAAAGCCCTGAAGT	[7]
	R	TATTCGGCCAAAGCGTATTC	
Reference genes			
GAPDH	F	GGAGTCAACGGATTTGGTCGTA	[6]
	R	TTCTCCATGGTGGTGAAGAC	
B2M	F	AGGCTATCCAGCGTACTCCA	N/A
	R	CGGATGGATGAAACCCAGACA	
ARF1	F	GTGTTCGCCAACAAGCAGG	
	R	CAGTTCCTGTGGCGTAGTGA	
PUM1	F	GGCGTTAGCATGGTGGAGTA	
	R	CATCCCTTGGGCCAAATCCT	
ChIP regions			
RARRES1 region A	F	TGCCCGGCTAATTTTTGTAT	[8]
	R	GCTCACGAGGTCAGGAGTTT	
Region B	F	CACTGTGCGAGGCAGATTTA	
	R	AACACTTGCTGCCTCCATTC	
Region C	F	CCAAGCATTAGGGCTGTGAT	
	R	GACTTCTCCCACCTCCACAG	
Region D & CTCF response element	F	CACTCCTTTTCCACGTTTCC	
	R	ATGCCGCATCCTAGCACTAA	
RARα response element	F	TTGGTCTGGGTTTCTGATTCTT	N/A
	R	CTCAATCTTGTGTGTGCTTGTG	

Supplementary Table S3: Patient cohort details

**Sample details**		**Sample size (N)**
ER status	Positive	52
	Negative	10
PR status	Positive	47
	Negative	15
HER2 status	Positive	11
	Negative	48
	Not determined	3
Stage	I	12
	IIA	22
	IIB	11
	IIIA	8
	IIIB	4
	IIIC	3
	IV	2
Lymphovascular Invasion	Present	33
	Absent	29
Distal metastasis	M0	32
	M1	2
	MX	28
Average age at diagnosis	(years)	55.1
