# Supplementary material for: Breast cancer subtype dictates DNA methylation and ALDH1A3-mediated expression of tumor suppressor RARRES1
Source: Oncotarget. 2016 Jun 6;7(28):44096–112. doi: 10.18632/oncotarget.9858 (PMC5190082; doi:10.18632/oncotarget.9858)
Supplement: Supplementary file 3 [file oncotarget-07-44096-s003.docx]

**Supplementary Table 1: Cell line subtypes and culture specifications**

| **Cell line** | **Hormone Receptor Status** | | | **Subtype** | **Reference** |  | **Cell culture specifications** | | | | | |
| --- | --- | --- | --- | --- | --- | --- | --- | --- | --- | --- | --- | --- |
|  | **ER** | **PR** | **Her2** |  |  |  | **Basal medium** | **Additives** | |  | **Conditions** | **Passaging** |
| HCC38 | - | - | - | claudin-low | [1] |  | RPMI-1640 | 10% FBS |  |  | 5% CO2 | 0.25% trypsin |
| BT-549 | - | - | - | claudin-low | [2] |  | RPMI-1640 | 10% FBS | 0.023 IU/mL human insulin |  | 5% CO2 | 0.25% trypsin |
| Hs 578T | - | - | - | claudin-low | [2] |  | DMEM | 10% FBS | 0.01 mg/mL bovine insulin |  | 5% CO2 | 0.25% trypsin |
| MDA-MB-231 | - | - | - | claudin-low | [2] |  | L-15 | 10% FBS |  |  | 0% CO2 | 0.25% trypsin |
| MDA-MB-436 | - | - | - | claudin-low | [2] |  | L-15 | 10% FBS | 10 ug/mL insulin, 16 ug/mL glutathione |  | 0% CO2 | scraped |
| MDA-MB-157 | - | - | - | claudin-low | [2] |  | L-15 | 10% FBS |  |  | 0% CO2 | 0.25% trypsin |
| HCC1395 | - | - | - | claudin-low | [1] |  | RPMI-1640 | 10% FBS |  |  | 5% CO2 | 0.25% trypsin |
| SUM159PT | - | - | - | claudin-low | [2] |  | F12 | 5% FBS | HEPES, 1 ug/mL hydrocortisone, 5 ug/mL human insulin |  | 5% CO2 | 0.25% trypsin |
| SUM1315MO2 | - | - | - | claudin-low | [2] |  | F12 | 5% FBS | 10 ng/mL EGF, HEPES, 5 ug/mL human insulin |  | 5% CO2 | 0.25% trypsin |
| HCC1937 | - | - | - | basal | [2] |  | RPMI-1640 | 10% FBS |  |  | 5% CO2 | 0.25% trypsin |
| HCC1143 | - | - | - | basal | [1] |  | RPMI-1640 | 10% FBS |  |  | 5% CO2 | 0.25% trypsin |
| MDA-MB-468 | - | - | - | basal | [2] |  | L-15 | 10% FBS |  |  | 0% CO2 | 0.25% trypsin |
| HCC70 | - | - | - | basal | [2] |  | RPMI-1640 | 10% FBS |  |  | 5% CO2 | 0.25% trypsin |
| HCC1806 | - | - | - | basal | [3] |  | RPMI-1640 | 10% FBS |  |  | 5% CO2 | 0.25% trypsin |
| HCC1187 | - | - | - | basal | [2] |  | RPMI-1640 | 10% FBS |  |  | 5% CO2 | mixed |
| BT-20 | - | - | - | basal | [2] |  | MEM | 10% FBS | NEAA, sodium pyruvate |  | 5% CO2 | 0.25% trypsin |
| SUM149PT | - | - | - | basal | [2] |  | F12 | 5% FBS | HEPES, 1 ug/mL hydrocortisone, 5 ug/mL human insulin |  | 5% CO2 | 0.25% trypsin |
| HCC1599 | - | - | - | basal | [2] |  | RPMI-1640 | 10% FBS |  |  | 5% CO2 | suspension |
| Du4475 | - | - | - | other | [3] |  | RPMI-1640 | 10% FBS |  |  | 5% CO2 | suspension |
| MDA-MB-453 | - | - | - | Her2 | [3] |  | L-15 | 10% FBS |  |  | 0% CO2 | 0.25% trypsin |
| SKBR3 | - | - | + | Her2 | [1] |  | DMEM | 10% FBS |  |  | 5% CO2 | 0.05% trypsin |
| BT474 | + | + | + | Her2/luminal | [1] |  | IMDM | 10% FBS |  |  | 5% CO2 | 0.25% trypsin |
| MCF-7 | + | + | - | luminal | [2] |  | DMEM | 10% FBS |  |  | 5% CO2 | 0.05% trypsin |
| T-47D | + | + | - | luminal | [2] |  | DMEM | 10% FBS |  |  | 5% CO2 | 0.05% trypsin |
| Hs578Bst |  |  |  | normal | [4] |  | IMDM | 10% FBS |  |  | 5% CO2 | 0.25% trypsin |
| MCF-10A |  |  |  | normal | [5] |  | DMEM/F12 | 5% horse serum | 20 ng/mL EGF, 0.5 mg/mL hydrocortisone, 10 ug/mL bovine insulin |  | 5% CO2 | 0.05% trypsin |
